# Build Your Bioprocess on a Solid Strain—β-Carotene Production in Recombinant *Saccharomyces cerevisiae*

**DOI:** 10.3389/fbioe.2019.00171

**Published:** 2019-07-18

**Authors:** Javiera López, Vicente F. Cataldo, Manuel Peña, Pedro A. Saa, Francisco Saitua, Maximiliano Ibaceta, Eduardo Agosin

**Affiliations:** ^1^Centro de Aromas and Sabores, DICTUC S.A., Santiago, Chile; ^2^Department of Chemical and Bioprocess Engineering, School of Engineering, Pontificia Universidad Católica de Chile, Santiago, Chile

**Keywords:** bioprocess, scale up, fermentation, *Saccharomyces cerevisiae*, β-carotene

## Abstract

Robust fermentation performance of microbial cell factories is critical for successful scaling of a biotechnological process. From shake flask cultivations to industrial-scale bioreactors, consistent strain behavior is fundamental to achieve the production targets. To assert the importance of this feature, we evaluated the impact of the yeast strain design and construction method on process scalability -from shake flasks to bench-scale fed-batch fermentations- using two recombinant *Saccharomyces cerevisiae* strains capable of producing β-carotene; SM14 and βcar1.2 strains. SM14 strain, obtained previously from adaptive evolution experiments, was capable to accumulate up to 21 mg/g_DCW_ of β-carotene in 72 h shake flask cultures; while the βcar1.2, constructed by overexpression of carotenogenic genes, only accumulated 5.8 mg/g_DCW_ of carotene. Surprisingly, fed-batch cultivation of these strains in 1L bioreactors resulted in opposite performances. βcar1.2 strain reached much higher biomass and β-carotene productivities (1.57 g/L/h and 10.9 mg/L/h, respectively) than SM14 strain (0.48 g/L/h and 3.1 mg/L/h, respectively). Final β-carotene titers were 210 and 750 mg/L after 80 h cultivation for SM14 and βcar1.2 strains, respectively. Our results indicate that these substantial differences in fermentation parameters are mainly a consequence of the exacerbated Crabtree effect of the SM14 strain. We also found that the strategy used to integrate the carotenogenic genes into the chromosomes affected the genetic stability of strains, although the impact was significantly minor. Overall, our results indicate that shake flasks fermentation parameters are poor predictors of the fermentation performance under industrial-like conditions, and that appropriate construction designs and performance tests must be conducted to properly assess the scalability of the strain and the bioprocess.

## Introduction

The improvement of experimental and analytical techniques, together with emerging synthetic biology tools, have enabled rapid and precise genetic manipulation of microorganisms for the industrial production of diverse compounds (Ajikumar et al., 2010; Paddon and Keasling, 2014; Meadows et al., 2016; Lian et al., 2018). These advances have greatly accelerated the construction and evaluation of promising microbial cell factories replacing traditional chemical synthesis processes, however, there are still many obstacles that difficult scaling lab-scale experiments up to economically attractive industrial bioprocesses (Yadav et al., 2012). In addition to low production titers, yields, and productivities, the often unpredictable physiology of microbial cell factories renders the scale-up process an expensive, time-consuming, labor-intensive task (Woolston et al., 2013; Wu et al., 2016). While much attention has been paid to the development of upstream operations (i.e., strain construction), seldom assessed under production conditions (i.e., fed-batch cultivations), the production performance is typically only evaluated in laboratory-scale batch cultures, which may greatly differ from the actual process behavior (Lee and Kim, 2015; Petzold et al., 2015; Gustavsson and Lee, 2016).

A family of interesting compounds with seemingly attractive scalability potential in yeast cell factories are carotenoids, particularly β-carotene (Mata-Gómez et al., 2014; Larroude et al., 2018). This C_40_ isoprenoid is widely used in the food and health industries as feed additive and/or nutraceutical (Mata-Gómez et al., 2014; Niu et al., 2017). A number of studies have reported high heterologous β-carotene production in shake flasks (Yamano et al., 1994; Verwaal et al., 2007; Li et al., 2013, 2017; Zhao et al., 2015) and bench-scale batch fermentations (Reyes et al., 2014; Olson et al., 2016). However, there is scarce data about the scalability of the bioprocess under more realistic production conditions (i.e., fed-batch mode). To the best of our knowledge, there is only one study reporting high β-carotene production in a recombinant yeast strain during fed-batch fermentations (achieving up to 20.8 mg/g_DCW_ total carotenoid content and 9.6 mg/L/h volumetric productivity) (Xie et al., 2015). However, analysis of the overall scalability of the generated strain and proposed bioprocess are still lacking.

In this work, we evaluated the impact of the design and construction method on the production performance and scalability potential of two engineered yeast strains. As a case study, we chose the heterologous production of β-carotene in *S. cerevisiae*. The evaluated strains were built following two radically different approaches; one transformed and evolved by adaptive evolution using oxidative stress as selective pressure for accumulating high amounts of β-carotene (SM14)—to date the highest carotenoid-accumulating yeast strain in test tubes (18 mg/g_DCW_) (Reyes et al., 2014) and batch fermenters (25 mg/g_DCW_) (Olson et al., 2016); and the other, constructed using the industrial CEN.PK2-1c strain as metabolic chassis and transformed employing state-of-the-art molecular biology tools (βcar1.2). In addition to the usual fermentation characterization in shake flasks, genomic stability assays and fed-batch culture experiments in bench-scale bioreactors were performed to assess the suitability of the strains for high and robust β-carotene production. Our results show that conclusions drawn from preliminary characterizations performed under settings different than the actual production conditions can be misleading and that rigorous evaluation of the producer strains should be conducted to properly assess the scalability of both, the strain and the bioprocess.

## Materials and Methods

### Plasmids Construction

Integrative plasmids needed for heterologous β-carotene production in the *S. cerevisiae* CEN.PK2-1c strains were constructed using Gibson assembly (Gibson, 2009). The carotenogenic enzyme genes (*crtE, crtYB*, and *crtI*) from *Xanthophyllomyces dendrorhous* were amplified by PCR, using genomic DNA from the *S. cerevisiae* SM14 strain as template. The catalytic domain of the truncated HMG-CoA reductase gene (*tHMG1*) was amplified by PCR using genomic DNA from the CEN.PK2-1c strain of *S. cerevisiae* as template. Backbone vectors were amplified by PCR, using the plasmid library developed by Mikkelsen et al. (2012) with primer pair homology to either TEF1 or PGK1 promoters, and to either ADH1 or tCYC1 terminators. All PCR products were gel-extracted to eliminate original vector residues. Purified PCR amplified vectors (100 ng) were mixed in a molar ratio depending on their length, following the manufacturer's instruction. DNA fragments were mixed with home-made Gibson master mix (5X isothermal mix buffer, T5 exonuclease 1 U/μL, Phusion DNA polymerase 2 U/μL, Taq DNA ligase 40 U/μL and Milli-Q purified water) until reaching 10 μL working volume. The mixture was incubated for 60 min at 50°C. Finally, the reaction mix was used to transform chemically competent *E. coli* TOP10 cells (ThermoFisher, USA). All vectors contained one marker gene (URA3, TRP1 or LEU2) depending on the parental strain auxotrophy.

### Strains

Two *S. cerevisiae* strains, SM14 and βcar1.2, were employed to compare their production performance and evaluate their genetic stability. The SM14 is a β-carotene hyper-producer strain derived from adaptive evolution experiments (Reyes et al., 2014). For the genomic stability evaluation, the SM14 was transformed with a 120-bp PCR product containing a 60-base-pair homology with the flanking regions of the URA3 gene, yielding a yeast strain with uracil auxotrophy (SM14-ΔURA3). In the case of βcar1.2 strain, the CEN.PK2-1c strain was the chassis employed for its incremental construction through βcar1 and βcar1.1 strains. A summary of the strains employed in this study and their genotype is shown in Table 1.

**Table 1:** Strains used in this study.

**Strain**	**Parental strain**	**Genotype**	**References**
SM14	S288c	MATa, P*_*TDH*3_-crtYB-*T*_*CYC*1_* P*_*TDH*3_-crtI-*T*_*CYC*1_* P*_*TDH*3_-crtE-*T*_*CYC*1_ ΔCTT1* *URA3*	Reyes et al., 2014
SM14-ΔURA3	SM14	ΔURA3	This study
CEN.PK2-1c		MATa, ura3-52, trp1-289, leu2-3, 112 his3Δ, can1Δ::cas9-natNT2	Euroscarf IMX672
βcar1	CEN.PK2-1c	(XI-5) P*_*TEF*1_-tHMG1*-T*_*ADH*1_* P*_*PGK*1_-crtYB*-T*_*CYC*1_* (XI-3) P*_*TEF*1_-crtI*-T*_*ADH*1_* P*_*PGK*1_-crtE-*T*_*CYC*1_ URA3*	This study
βcar1.1	βcar1	(X-2) P*_*TEF*1_-tHMG1*-T*_*ADH*1_* P*_*PGK*1_-tHMG1X.d*.-T_*CYC*1_ *LEU2*	This study
βcar1.2	βcar1.1	(XI-1) P*_*PGK*1_-crtYB*-T*_*CYC*1_* P*_*TEF*1_-crtI*-T*_*ADH*1_ TRP1*	This study

Transformations were performed using lithium acetate/single-stranded DNA carrier/PEG procedure (Gietz and Woods, 2002) and SC proper plates for transformants selection. Finally, correct cassette integration into the specific loci was tested by colony PCR, and carotenoid production was evaluated in YPD medium at 30°C after 72 h (see *Carotenoid extraction and analysis*).

### Shake Flask Cultures and Genetic Stability Evaluation

For ethanol, glucose, acetate, biomass and total carotenoids quantification, a single colony was picked from YPD or CSM (with or without auxotrophy) agar plates, subcultured in tubes overnight at 30°C and 160 rpm in a rotary shaker incubator in 3 mL of YPD medium. On the next day, the optical density at 600 nm (OD_600_) of each culture tube was measured (see *Biomass determination*). The content of the tubes was then transferred to a 250 mL baffled shake flask with 50 mL final culture volume at an initial OD600 of 0.1 in YPD medium. Culture samples were periodically collected for biomass, extracellular metabolites and carotenoids quantification.

Genetic stability was evaluated in 72-h batch cultures, following the same shake-flask cultivation protocol. After 72 h, an aliquot of each culture (previously diluted to 1 mL at OD600 = 10) was diluted 10,000-fold and 100 μL were plated in YPD. Furthermore, the kinetics of carotenogenic gene loss after several generations was determined in exponential phase cultures. For this purpose, the cultures were started at OD600 of 0.1 in YPD until late exponential phase (12 h) and then diluted again to an OD600 of 0.1 (twice per day). Samples at different cultivation times were diluted and plated in YPD. After 4 days at 30°C, orange and white colonies were counted from all plates. The number of generations was calculated according to the duplication time of the cultures.

### Fermentation Conditions and Culture Media

Fed-batch cultures were performed in 1-L in-house bioreactors equipped with a condenser, a stirrer and two Rushton turbines operated with brushless DC motors (Oriental Motor, Japan). A SIMATIC PCS7 control system (Siemens, Germany) was used to monitor and control the cultivations at 30°C, pH = 5.0 and dissolved oxygen above 2.8 mg/L. These culture conditions were employed throughout the entire study for all fermentations. Aerobiosis was maintained with a modified split-range control scheme varying the agitation, air and pure oxygen gas flows (Cárcamo et al., 2014). Briefly, as the oxygen demand increases, the control scheme first increases the agitation from 200 to 500 rpm, then the air flow from 0.3 to 1 L/min, and finally, if needed, pure oxygen gas flow from 0.05 to 1 L/min with the concomitant decrease in air flow, thereby maintaining the total gas inflow constant.

The batch medium used in the bioreactor cultivations of both strains contained (per liter): 20 g glucose, 15 g ammonium sulfate, 500 mg leucine, 160 mg histidine, 100 mg tryptophan, 4 g KH_2_PO_4_, 1.2 g MgSO_4_·7H_2_O, and 150 mg NaCl. The medium was supplemented with 15 mL/L of a vitamin solution, 3 mL/L of a trace solution, 0.75 mL/L of a CaCl_2_·2H_2_O solution at 40 g/L, and 0.75 mL/L of a FeSO_4_·7H_2_O solution at 4.2 g/L. The trace solution is composed of 3.3 g/L of zinc sulfate heptahydrate, 2 g/L of cobalt chloride hexahydrate, 3.3 g/L of manganese sulfate monohydrate, 4.67 g/L of copper sulfate pentahydrate, 2 g/L of boric acid, 0.2 g/L of potassium iodide, 0.46 g/L of molybdic acid sodium salt dihydrate and 8 g/L of EDTA. In addition, the vitamin solution consists of 0.05 g/L of D-Biotin, 5 g/L of calcium pantothenate, 3.75 g/L of nicotinic acid, 40 g/L of myo-inositol, 1 g/L of thiamine- HCl, 2.5 g/L of Pyridoxine-HCl, 0.02 g/L of p-aminobenzoic acid, 1 g/L of riboflavin and 0.02 g/L of folic acid. Finally, in the case of the fed-batch cultures, the fed-batch feeding contained (per liter): 450 g glucose, 15 g KH_2_PO_4_, 5.5 g MgSO_4_·7H_2_O, and 15 mL/L casamino acids solution at 75 g/L. The fed-batch feeding was supplemented with 15 mL/L and 9 mL/L of the previous vitamin and trace solutions, respectively, 1.35 mL/L of CaCl_2_·2H_2_O solution at 400 g/L, and 1.35 mL/L of a FeSO_4_·7H_2_O solution at 84 g /L. Both vitamin and trace solutions were filter-sterilized before their use in all the mentioned media.

### Batch Cultures

A single colony from a working plate was cultured in 3 mL YPD medium in a pre-inoculum tube at 30°C for 10–14 h (overnight). On the next morning, 1 mL was cultured in a shake flask with 20 mL YPD medium for 8 h. Batch cultures in bioreactors were inoculated to a final concentration of OD600 of 0.1. Fermentation conditions were the same as the previous section. Culture samples were periodically collected every 2–3 h for biomass, extracellular metabolites and carotenoids quantification. Batch fermentations were stopped when all major carbon sources were exhausted (i.e., glucose, ethanol and acetic acid).

### Fed-Batch Cultures

**Figure 1:**
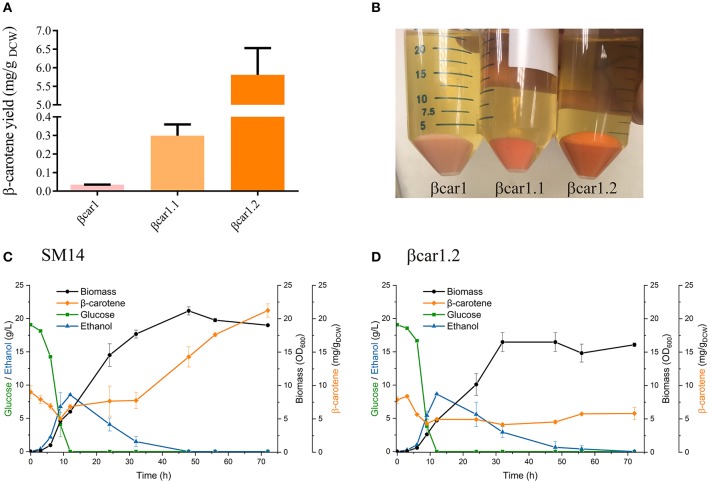
Shake flask performance of *Saccharomyces cerevisiae* βcar and SM14 strains. **(A)** β-carotene to biomass yield for βcar strains after 72 h shake flask cultures. **(B)** Pellets of βcar strains in shake flask cultures after 72 h. **(C)** SM14 strain performance in shake flask culture after 72 h. **(D)** βcar1.2 strain performance in 72 h shake flask cultures.

Fed-batch cultures were fed following an exponential feeding (Equation 1, 2) with an exponentially decreasing specific growth rate set (μ_set_) (Equation 3). Both *S. cerevisiae* strains (SM14 and βcar1.2) followed the same feeding profile, albeit with different initial parameters, depending on the fermentation profile observed in the shake flask cultivations. As both strains behaved differently in these cultures (refer to ethanol and β-carotene production, Figures 1C,D), and in order to provide a fair evaluation, we employed slightly different feeding parameters so that the strains could be more easily compared. For both strains, the feeding started after both glucose and ethanol were depleted using an initial fixed specific growth rate. The exponentially decreasing specific growth rate feeding strategy was started once the biomass concentration reached approx. an OD_600_ of 60. The general feeding profile is described by the conventional exponential formula (Villadsen and Patil, 2007),

(1) $$\begin{array}{rcl} F\left(t\right) & = & F_{\text{in}}\cdot \exp\left\{\mu_{\text{set}}\left(t\right)\cdot t\right\} \end{array}$$

(2) $$\begin{array}{rcl} F_{\text{in}} & = & \mu_{\text{set}}{\left(XV\right)}_{\text{in}}/\left(Y_{\text{sx}}\cdot S_{\text{in}}\right) \end{array}$$

where *F*_0_ describes the initial feeding rate. The parameters of the latter formula correspond to the total amount of biomass in the reactor at the beginning of the feeding (*XV*)_in_, the biomass yield on glucose (*Y*sx), and the glucose concentration in the feed (*S*in). In the case of μ_set_(*t*), this parameter is strain-dependent and was defined by Equation 3 with values between 0.1–0.13 h^−1^ for μ_init_ and 0.03 h^−1^ for μ_end_.

(3) $$\begin{array}{r} \mu_{\text{set}}\left(t\right)=\left(\mu_{\text{init}}-\mu_{\text{end}}\right)\cdot \text{exp}\left\{-\mu_{\text{end}}\left(t\right)\cdot t\right\}+\mu_{\text{end}} \end{array}$$

Finally, culture samples were periodically collected for biomass, extracellular metabolites and carotenoids quantification.

### Biomass Determination

Biomass concentration was determined by optical density (OD600) using an UV-160 UV-visible spectrophotometer (Shimadzu, Japan). Biomass concentration was estimated using the linear relationship: 1 OD_600_ = 0.4 g/L determined experimentally.

### Extracellular Metabolite Quantification

Culture samples were centrifuged at 10,000 rpm for 3 min and the supernatant stored at −80°C for metabolite analysis. Extracellular glucose, ethanol and acetic acid concentrations were quantified in duplicate by High-Performance Liquid Chromatography (HPLC) as detailed in Sánchez et al. (2014).

### Carotenoid Extraction and Analysis

For each sample, 8 mg of biomass were pelleted into 2 mL Eppendorf tubes and the supernatant was discarded. Four hundred microliter of acid-washed glass beads (Sigma Aldrich, USA) and 1 mL of hexane were then added for cell disruption and carotenoid extraction from the cell membranes. Cells were disrupted at room temperature in a BeadBug6 cell homogenizer (Benchmark Scientific, USA) using a program consisting in 4 cycles of 90 s of disruption at 3,700 rpm, followed by a 10-s rest. Cell lysate was then centrifuged and the supernatant (hexane) was stored at −80°C until further analysis. Carotenoid quantification was performed by measuring the absorbance at 453 nm of the hexane extracts, and then converted into concentrations using a standard curve of β-carotene ranging from 0.5 to 10 mg/L.

## Results

### Construction of βCar Yeast Strains

A series of β-carotene-producing yeast strains (βcar) were constructed using a CEN.PK strain as host cell. Integration of carotenogenic genes in stable constructs was achieved using two different promoters (in a bidirectional arrangement) and two different terminators. The integration of the three carotenogenic genes (*crtE, crtYB* and *crtI*), together with the tHMG1 gene, resulted in a strain (βcar1) that generated faint orange colonies. This strain was capable of accumulating 0.034 mg/g_DCW_ β-carotene in shake flasks after 72 h of incubation. This initial βcar strain was further optimized by integrating two extra copies of the tHMG1 gene (βcar1.1 strain) and, then, by adding an extra copy of the *crtYB* and *crtI* genes (final βcar1.2 strain) (Figures 1A,B). The βcar1.1 and βcar1.2 strains accumulated, respectively, 0.3 mg/g_DCW_ and 5.8 mg/g_DCW_ β-carotene in shake flasks after 72 h of incubation (Figure 1A).

### Strain Performance in Shake Flask Cultures

Shake flask batch cultures of the βcar1.2 and SM14 displayed similar fermentation profiles during the first 24 h incubation (Figures 1C,D). Both, glucose consumption and ethanol production behaved similarly during the first 12 h, and there were no substantial differences in β-carotene levels before 24 h. In addition, the maximum specific growth rate reached similar values during this time (0.43 h^−1^ for SM14 and 0.40 h^−1^ for βcar1.2), consistent with the profiles of the main fermentation substrates and products. However, this trend was not maintained throughout the cultivation. While βcar1.2 showed an almost constant β-carotene concentration after the first 24 h of cultivation (~5 mg/g_DCW_), SM14 quadrupled this value, rising from 7.6 mg/g_DCW_ at 24 h, to 21 mg/g_DCW_ after 72 h cultivation (Figures 1C,D). Interestingly, the SM14 strain displayed an important increase in the specific β-carotene production rate upon reaching the stationary phase, which was not replicated by the βcar1.2 strain. Altogether, the final β-carotene titer of the SM14 strain reached 159.6 mg/L by the end of the shake flask fermentation, roughly four times higher than the βcar1.2 titers.

### Strain Performance in Fed-Batch Cultures

**Figure 2:**
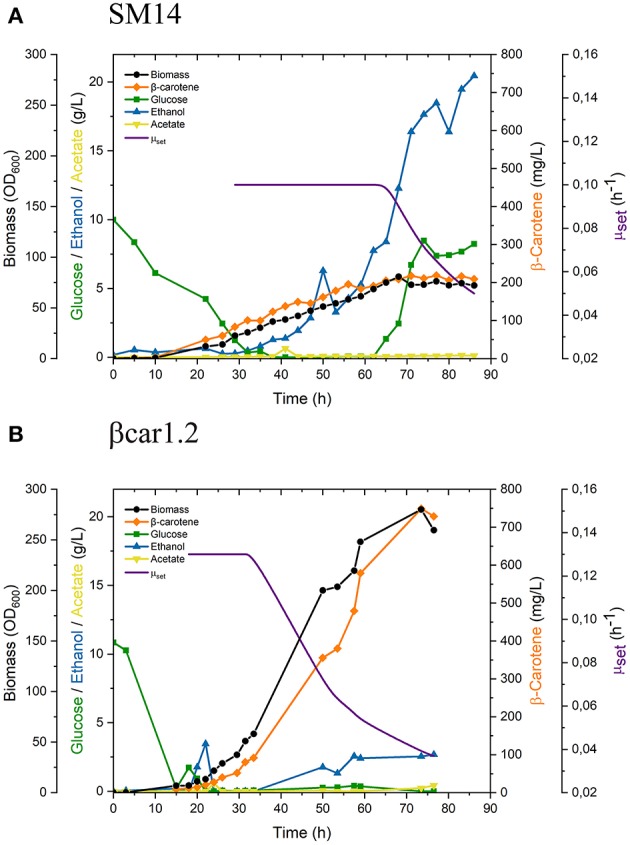
Fed-batch fermentation performance of **(A)** SM14 and **(B)** βcar1.2 strains. Exponential decreasing feeding started when the culture reached a biomass concentration of OD_600_ = 60.

SM14 strain was grown in fed-batch mode to evaluate its performance under production conditions (Figure 2A). Once the glucose from the batch phase was depleted (after approx. 26 h of cultivation), the feeding was started according to Equation 1 with a constant specific growth rate set of 0.1 h^−1^. After 65 h of cultivation and upon reaching 60 OD_600_, the exponentially decreasing specific growth rate set protocol was initiated using an initial μ_init_ of 0.1 h^−1^ and a final μ_end_ of 0.03 h^−1^ to be reached within the next 24 h. This change in the feeding policy was performed to enable reaching higher cell densities using a more conservative feeding strategy.

After 68 h cultivation, biomass growth stopped, which was consistent with the glucose (2.4 g/L) accumulation (Figure 2A). At this point, biomass concentration reached 32.3 g/L (72.6 OD_600_, biomass volumetric productivity q_X_ = 0.475 g_DCW_/L/h) and the carotenoid content of the cells was 6.48 mg/g_DCW_, yielding a total carotenoid titer of 209 mg/L (carotenoid volumetric productivity q_C_ = 3.07 mg/L/h) (Table 2).

**Table 2:** Fermentation parameters in fed-batch cultivations at 68 h cultivation for the two *S. cerevisiae* strains of this study.

**Parameter**	**SM14 strain**	**βcar1.2 strain**
Biomass titer (g/L)	32.3	107.1
Biomass productivity (g/L/h)	0.475	1.576
β-carotene titer (mg/L)	209.0	739.6
β-carotene productivity (mg/L/h)	3.07	10.88
β-carotene to biomass yield (mg/g)	6.48	6.91

Both biomass and carotenoid concentrations plateaued thereafter. For instance, after 77 h of cultivation, 30.5 g/L of biomass (76.2 OD_600_, q_X_ = 0.396 g_DCW_/L/h) with a carotenoid content of 7.18 mg/g_DCW_ were achieved, yielding a total carotenoid titer of 218 mg/L (carotenoid volumetric productivity q_C_ = 2.83 mg/L/h). Finally, ethanol accumulation started as soon as the constant exponential feed was initiated, reaching concentrations of 12.3 g/L at 68 h cultivation and >18 g/L after 77 h.

Similar to SM14 strain, the engineered βcar1.2 strain, was evaluated in fed-batch fermentations under production conditions (Figure 2B). Once the glucose and ethanol from the batch phase were depleted, the feeding was started according to Equation 1 with a constant specific growth rate set of 0.13 h^−1^. A slightly higher μ_set_ was employed in this case as this strain showed a higher μ_critical_, as determined in preliminary fermentations. Again, upon reaching 60 OD_600_ (approx. after 33 h cultivation), the feeding policy was changed to the exponentially decreasing policy with an initial μ_init_ of 0.13 h^−1^ and a final μ_end_ of 0.03 h^−1^.

After 68 h of fed-batch cultivation, the βcar1.2 strain reached a biomass concentration of 107.1 g_DCW_/L (267.9 OD_600_, biomass volumetric productivity q_X_ = 1.576 g_DCW_/L/h) with a carotenoid content of 6.91 mg/g_DCW_, overall yielding a total carotenoid titer of 739.6 mg/L (carotenoid volumetric productivity q_C_ = 10.88 mg/L/h). Later fermentation results were consistent with this data. After 77 h of fermentation, 103.8 g/L of biomass were produced, yielding a volumetric productivity of 1,34 g_DCW_/L/h. Importantly, the biomass productivity of the βcar1.2 strain significantly outperformed (~3.5-fold higher) the previous results for the SM14 strain (Table 2). Likewise, the carotene productivity of the βcar1.2 strain was 3.3-fold higher than that reached by the SM14 strain. After 77 h cultivation, a total carotenoid titer of 729 mg/L (7 mg/g_DCW_ carotene yield) was achieved with this strain (Table 2).

### Strain Stability Analysis

**Figure 3:**
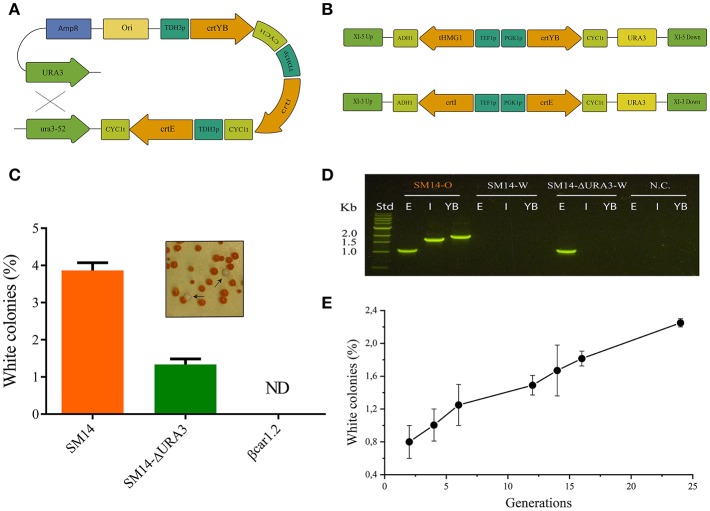
Stability of βcar and SM14 strains. **(A)** General scheme of the possible homologous recombination of the SM14 carotenogenic expressing construct. **(B)** Architecture for the expression of the carotenogenic genes in βcar strains. Integration stability is based in two recombination sites (UP and DOWN) and the use of two different promoters and terminators. **(C)** Percentage of white colonies observed after 72 h liquid cultures in strains with the original construct (SM14) and with the deletion of the URA3 gene (SM14-ΔURA3). ND: not detected. **(D)** Detection of carotenogenic genes by agarose gel of genomic PCR products from SM14 orange phenotype (SM14-O), SM14 white phenotype (SM14-W) and SM14-ΔURA3 white phenotype (SM14-ΔURA3-W). E, I, YB refers to *crtE, crtI* and *crtYB* respectively. Std: standard DNA ladder. NC: negative control. **(E)** Percentage of white colonies observed during exponential cultures of SM14.

In order to determine if genetic instability had a significant impact on β-carotene productivity, we evaluated the rate of generation of white cells in the yeast population, which is indicative of carotenogenic genes loss. The SM14 strain showed a 3.9% of white colonies when 72-h shake flask cultures were plated, indicating an intrinsic genetic instability of the carotenogenic construct (Figure 3C). Since this strain contained repeated URA3 loci (URA3 and ura3-52) - flanking the carotenogenic genes -, and the same TDH3 promoters and CYC1 terminators for the latter genes, homologous recombination between direct repeat sequences, e.g., URA3 and ura3-52, had likely occurred (Figure 3A). In order to avoid possible recombination of the construct and improve the genetic stability of this strain, the URA3 marker was deleted and a new strain, SM14-ΔURA3, was generated. Targeted deletion of the marker decreased the white colony number in YPD plates from 3.9% to 1.3% (Figure 3C). However, the SM14-ΔURA3 still showed carotenogenic gene loss, suggesting an inter-promoter or inter-terminator homologous recombination. Genomic PCR results further supported the loss of all carotenogenic genes in the white phenotype of the SM14 and the loss of two genes (*crtI* and *crtYB*) in the case of the white phenotype of the SM14-ΔURA3 (Figure 3D). These results are in concordance with the recombination scheme proposed, i.e., URA3 loci recombination in SM14 and inter-promoter recombination in SM14-ΔURA3, although other recombinations cannot be discarded (e.g., inter-promoter recombination in SM14).

Figure 3E shows a more detailed analysis of the genetic stability of the SM14 strain. We observed an increase in the proportion of white cells as the number of generations progressed, consistent with a rate of appearance of 0,06% white cells/generation. Notably, after 15 generations (close to the duration of a fed-batch cultivation), the proportion of white cells reached ~1.7 %.

## Discussion

### Design and Construction Strategy of the Engineered Yeast Strains Determines Fermentation Performance in Fed-Batch Cultures

Depending on fermentation phase, Reyes et al. (2014) reported different β-carotene to biomass yields for the SM14 strain in batch cultures: 6 mg/g_DCW_ during the glucose consumption phase, and 15 mg/g_DCW_ during the ethanol consumption phase. These results were in line with our findings for the SM14 strain behavior in shake flasks (5-7 mg/g_DCW_ during the glucose consumption phase, and 21 mg/g_DCW_ after the ethanol consumption phase, Figure 1C). However, these results were not scalable in fed-batch cultures for this strain (Figure 2A). In spite of employing a (conservative) μ decreasing strategy, the SM14 strain was unable to assimilate glucose without producing ethanol, even at low specific growth rates. In fact, ethanol accumulated to such high levels that growth was completely arrested (Figure 2A).

Previous DNA microarrays analysis for the SM14 strain showed that several genes involved in mitochondrial respiration and electron transport were downregulated, relative to its parental strain (e.g., SDH1, COX4, QCR9 and SDH3; Reyes et al., 2014). Apparently, β-carotene accumulation was not sufficient to overcome the oxidative stress from the oxygen peroxide shocks used to evolve the SM14 strain; thus, SM14 cells might reduce their mitochondrial oxidative capacity to lower the generation of radical oxygen species (ROS). These transcriptional changes are consistent with the enhanced Crabtree effect observed in our fed-batch fermentations. The latter suggests that impaired mitochondrial respiration is the main cause for the poor fermentation performance of the SM14 strain.

Since the SM14 strain has a poor oxidative capacity, other feeding policies could be explored in order to increase the biomass productivity. Nevertheless, the lack of knowledge of the complete genetic background of SM14 renders this strain unsuitable for its transfer to larger scales. In contrast to SM14, the βcar1.2 strain exhibited a more robust and satisfactory performance in the fed-batch cultivations (Figure 2B). Despite accumulating a quarter of the concentration of β-carotene compared to SM14 in shake flask cultures after 72 h (Figures 1C,D), the βcar1.2 strain greatly surpassed the production performance of SM14 in bioreactors (Table 2). In fed-batch cultures, βcar1.2 exhibited a high oxidative rate, consistent with a fully oxidative metabolism on glucose and the high biomass and β-carotene productivities achieved. These results clearly illustrate that the strain performance must be evaluated in a proper setting, such that initially modest but more robust producers are not discarded early.

### Gene Integration Architecture Affects Strain Stability

SM14 strain was constructed using the YIPlac211 YBIE plasmid reported by Verwaal et al. (2007), which has the classic features of an integrating plasmid for yeast, i.e., it possesses a URA3 marker that also serves as recombination site for integration into the ura3-52 locus of the auxotrophic strain. As a consequence of a unique recombination event, the vector containing the carotenogenic construct is integrated and flanked by the URA3 loci (URA3 and ura3-52). Since these genes have sufficient sequence identity, direct repeat recombination and loss of the whole construct may occur. This can even happen in a selective medium (drop-out without uracil), as the recombination event can leave the URA3 allele instead of the ura3-52 (Figures 3A,B). Based on the time-course stability analysis up to 24 generations (Figure 3E), we note, however, that genetic instability of the SM14 strain does not heavily impact the fed-batch culture performance in the relevant time-scale (ca. 15 generations). This is also supported by the β-carotene production profile in the fed-batch cultivation that shows a proportional increase of carotene with biomass (Figure 2A). However, the genetic instability is still a disadvantage in pre-bioreactor stages considering that it introduces practical difficulties (e.g., selection of pigmented colonies) when handling the strain before bioreactor cultures. Lange and Steinbüchel (2011) reported that episomal expression of the same carotenogenic construct led to an entire plasmid loss due to segregational and structural instabilities, even when grown in selective medium. In this sense, integrating plasmids with one recombination site or repeated sequences are better choices over episomal counterparts. Even so, the stability of classical integrating plasmids like YIp was shown to be unsatisfactory to arrive at a robust strain for bioproduction (Figure 3).

To avoid the above stability issues, we built a new β-carotene strain producer, using the plasmid set developed by Mikkelsen et al. (2012). In these plasmids, the target genes are flanked by two different sequences for integration, preventing excision of the construct by homologous recombination. Moreover, the use of two different promoters and two different terminators further decreases the probability of gene loss. Consistent with these features, white colonies were not observed neither in βcar1.2 shake flask cultivations nor in stability studies, even in the absence of a selective pressure (medium with uracil), confirming the high stability of the genomic construct (Figures 3B,C).

## Conclusion

Robust fermentation performance is critical for the development and satisfactory scale up of biotechnological processes. Strain evaluation under realistic production conditions is critical to ensure appropriate process behavior. Here, we have compared the β-carotene production of two yeast strains built following radically different strategies. Our results indicated that the initially most promising evolved strain performed poorly under fed-batch production conditions compared to the conventionally-built strain. These results highlight the impact of the methodology employed for constructing and screening superior strains and its subsequent scale up. Particularly, the adaptive laboratory evolution impacted on the general microbial physiology, while the construct architecture affected the genetic stability of the strain, rendering a poorly scalable producer under production conditions. In contrast, the conventionally-built strain performed robustly, achieving higher biomass and β-carotene productivity in fed-batch cultivations. Overall, this work underscores the importance of carefully choosing the strain construction strategy and its optimization method taking into consideration the end goal. While some strains may perform well in batch cultures at small scales, and could be very useful for gene screening purposes, they may not necessarily perform adequately under production conditions. To this end, evaluation of a satisfactory production performance is a must and by no means can be extrapolated.

## Author Contributions

JL and VC constructed the strains. JL and MP performed shake flask cultures of the strains. VC designed and carried out the stability experiments. MP, FS, and MI carried out the fed-batch fermentation experiments in the bioreactors. JL, VC, MP, and PS analyzed the data. JL, VC, MP, FS, and PS participated in design, coordination of the study, and draft the manuscript. EA supervised the whole research and revised the manuscript. All authors read and approved the final manuscript.

### Conflict of Interest Statement

JL and FS were employed by company DICTUC S.A. EA is an advisor for DICTUC S.A. The remaining authors declare that the research was conducted in the absence of any commercial or financial relationships that could be construed as a potential conflict of interest.
